# Supplementary material for: A fault-intrusion-tolerant system and deadline-aware algorithm for scheduling scientific workflow in the cloud
Source: PeerJ Comput Sci. 2021 Nov 2;7:e747. doi: 10.7717/peerj-cs.747 (PMC8576554; doi:10.7717/peerj-cs.747)
Supplement: Supplemental Information 6 [file peerj-cs-07-747-s006.zip › Myproject1.1/docs/org/workflowsim/clustering/ClusteringInterface.html]

ClusteringInterface


JavaScript is disabled on your browser.


- Overview
- Package
- Class
- Use
- Tree
- Deprecated
- Index
- Help

- Prev Class
- Next Class

- Frames
- No Frames

- All Classes

- Summary:
- Nested |
- Field |
- Constr |
- Method

- Detail:
- Field |
- Constr |
- Method


org.workflowsim.clustering

## Interface ClusteringInterface

- All Known Implementing Classes:
  :   BalancedClustering, BasicClustering, BlockClustering, HorizontalClustering, VerticalClustering

  ---

    

  ```
  public interface ClusteringInterface
  ```

  The ClusteringInterface for all clustering methods

  Since:
  :   WorkflowSim Toolkit 1.0

- - ### Method Summary

    Methods

    | Modifier and Type | Method and Description |
    | --- | --- |
    | `java.util.List<Job>` | `getJobList()` get job list. |
    | `java.util.List<File>` | `getTaskFiles()` get all the task files. |
    | `java.util.List<Task>` | `getTaskList()` get task list. |
    | `void` | `run()` the main function. |
    | `void` | `setTaskList(java.util.List<Task> list)` set the task list. |

- - ### Method Detail


    - #### setTaskList

      ```
      void setTaskList(java.util.List<Task> list)
      ```

      set the task list.


    - #### getJobList

      ```
      java.util.List<Job> getJobList()
      ```

      get job list.


    - #### getTaskList

      ```
      java.util.List<Task> getTaskList()
      ```

      get task list.


    - #### run

      ```
      void run()
      ```

      the main function.


    - #### getTaskFiles

      ```
      java.util.List<File> getTaskFiles()
      ```

      get all the task files.


- Overview
- Package
- Class
- Use
- Tree
- Deprecated
- Index
- Help

- Prev Class
- Next Class

- Frames
- No Frames

- All Classes

- Summary:
- Nested |
- Field |
- Constr |
- Method

- Detail:
- Field |
- Constr |
- Method
